# Sequential Screening Strategy in Early, Middle, and Late Pregnancy in Women at High Risk of Hyperglycemia

**DOI:** 10.3389/fendo.2022.829388

**Published:** 2022-06-06

**Authors:** Yi Xu, Qiang Wei, Li Zhang, Mei-fan Duan, Yue-mei Wang, Nan Huang

**Affiliations:** ^1^ Department of Obstetrics and Gynecology, Key Laboratory of Birth Defects and Related Diseases of Women and Children of the Ministry of Education, West China Second University Hospital, Sichuan University, Chengdu, China; ^2^ Department of Obstetrics and Gynecology, Women and Children’s Hospital of Chongqing Medical University, Chongqing, China

**Keywords:** pregnancy, hyperglycaemia, screening strategy, risk factor, oral glucose tolerance

## Abstract

**Background:**

Hyperglycaemia in pregnancy (HIP) is closely associated with short- and long-term adverse fetal and maternal outcomes. However, the screening and diagnostic strategies for pregnant women with risk factors for HIP are not set. This prospective study aimed to explore a screening strategy for women at high risk for HIP.

**Methods:**

A total of 610 pregnant women were divided into experimental (n=305) and control (n=305) groups. Pregnant women underwent a 75-g OGTT in early (<20 weeks), middle (24–28 weeks), and late pregnancy (32–34 weeks) in the experimental group and only in middle pregnancy in the control group. The general conditions, HIP diagnosis, and perinatal outcomes of the two groups were compared.

**Results:**

In the experimental group, HIP was diagnosed in 29.51% (90/305), 13.44% (41/305), and 10.49% (32/305) of patient in early, middle, and late pregnancy, respectively. The total HIP diagnosis rate was significantly higher in the experimental group (53.44% vs. 35.74%, P<0.001). Multivariate logistic regression analysis revealed that previous gestational diabetes mellitus (GDM) (odds ratio, OR=9.676, P<0.001), pre-pregnancy body mass index (BMI) ≥23 kg/m^2^ (OR=4.273, P<0.001), and maternal age ≥35 years (OR=2.377, P=0.010) were risk factors for HIP diagnosis in early pregnancy. Previous GDM (OR=8.713, P=0.002) was a risk factor for HIP diagnosis in late pregnancy. No significant differences in perinatal clinical data were observed between the experimental and control groups. The gestational age at delivery was significantly earlier in the experimental subgroup with early-HIP than in the experimental and control subgroups with normal blood glucose (NBG). The weight gain during pregnancy was lower in the experimental early-HIP, middle-HIP, and control NBG subgroups.

**Conclusions:**

We recommend sequential screening in early and middle pregnancy for high-risk pregnant women with maternal age ≥35 years or pre-pregnancy BMI ≥23 kg/m^2^, and in early, middle, and late pregnancy for high-risk pregnant women with a previous history of GDM.

**Trial Registration:**

This study was registered in the Chinese Clinical Trial Registry (no. ChiCTR2000041278).

## Introduction

Hyperglycaemia in pregnancy (HIP) is a common complication during pregnancy and is closely associated with short- and long-term adverse fetal and maternal outcomes. Hyperglycaemia first identified in pregnancy may be categorized into gestational diabetes mellitus (GDM) and Diabetes in pregnancy (DIP). The global incidence of GDM has increased due to a considerable increase in the incidence of type 2 DM consequent to poor lifestyle choices, an update to the diagnostic criteria for GDM, and emphasis on GDM screening (1–3). Screening, diagnosing, and treating HIP are therefore particularly important. Nonetheless, despite the continuous progress of HIP research in recent years worldwide, scholars have yet to reach a consensus on various aspects.

In 2010, the International Association of Diabetes and Pregnancy Study Groups (IADPSG) proposed a new standard for diagnosing GDM based on the results of the Hyperglycemia and Adverse Pregnancy Outcome (HAPO) study (4). There are multiple screening schemes and diagnostic standards, and there are controversies concerning the diagnostic threshold and the merits and demerits of the one-step and two-step methods. The screening and diagnostic strategies for pregnant women without risk factors for HIP are not set, and no conclusion has been drawn for high-risk pregnant women. In view of the compromise between the diagnostic time for GDM and the potential for subsequent treatment and risk control, guidelines usually recommend that HIP screening should be conducted between 24 and 28 weeks of pregnancy, and that high-risk women should be screened during their first antenatal visit or early pregnancy. These early screening procedures are mainly intended to detect women with prediabetes or diabetes before pregnancy early on, rather than to predict those who will develop GDM in late pregnancy. Nevertheless, the available evidence suggests that GDM may be present before the recommended time for screening, particularly among high-risk women such as those with prior GDM or obesity (5, 6). When screening high-risk pregnant women in early pregnancy, the blood glucose levels sometimes fall between the diagnostic criteria for routine screening in early pregnancy and those for non-pregnant adults with diabetes, which has been referred to as early GDM (7). There is no consensus on the diagnostic threshold for early GDM. The World Health Organization (WHO) and the International Federation of Gynecology and Obstetrics (8) advocate that the same blood glucose threshold may be used to diagnose GDM at any stage of pregnancy. However, most guidelines do not seem to agree, and it appears that some guidelines deliberately avoid this.

In middle and late pregnancy, insulin-antagonistic substances such as tumor necrosis factor, placental prolactin, and leptin increase among pregnant women; hence, insulin sensitivity in pregnant women decreases with an increase in gestational age. In pregnant women with limited insulin secretion, this physiological change cannot be compensated, resulting in high blood glucose levels (9). This suggests that pregnant women with normal blood glucose levels during routine screening in middle pregnancy may still have high blood glucose levels in late pregnancy. Nonetheless, the current guidelines have few relevant recommendations for HIP screening after 28 weeks of pregnancy, and relevant research is lacking.

Maternal age ≥35 years, being overweight or obese before pregnancy, history of GDM, and polycystic ovary syndrome (PCOS) are considered high-risk factors for HIP. However, there are few study investigated the incidence of HIP and perinatal outcomes in high-risk pregnant women, particularly in the last 10 years after the release of the new IADPSG standard. Research on HIP screening programs for high-risk pregnant women only focuses on increasing early screening in early pregnancy, and studies on supplementary screening in late pregnancy are scarce. Moreover, studies in which sequential HIP screening is conducted in early, middle, and late pregnancy are even scarcer.

Our hospital is a critical care centre in southwest China and caters to multiple pregnant women with high-risk factors, including maternal age ≥35 years, being overweight, and obesity. Over the last 5 years, the incidence of HIP in our hospital’s obstetrics department was 25.28% (13893/54952). In the study, we aimed to find a HIP screening strategy (sequential screening in early, middle, and late pregnancy) for high-risk pregnant women taking advantage of regional (i.e., high incidence of HIP) and hospital resources (i.e., high proportion of high-risk pregnant women). Therefore, we analyzed the diagnosis rate of HIP between two screening strategies, the diagnostic basis of HIP diagnosis in different screening periods, and factors contributing to a diagnosis of HIP during early and late pregnancy, and comparison of perinatal outcomes between two screening strategies.

## Materials and Methods

### Study Subjects

A total of 610 pregnant women who had undergone antenatal examination and delivery at the West China Second University Hospital of Sichuan University from July 2017 to June 2019 were alternately divided into the experimental (*n*=305) and control (*n*=305) groups according to the chronological order of the first antenatal visit. The inclusion criteria were one or more of the following factors: (1) family history of diabetes (first-degree relatives with type 2 DM); (2) pre-pregnancy body mass index (BMI) ≥23 kg/m^2^; (3) maternal age ≥35 years; (4) PCOS before pregnancy; (5) impaired fasting glucose (IFG) or impaired glucose tolerance (IGT) before pregnancy; (6) history of GDM in previous pregnancy; and (7) history of macrosomia. The exclusion criteria were: (1) diagnosis of type 1 or 2 DM or other glucose and lipid metabolism diseases before pregnancy; (2) combination with serious internal and surgical diseases; (3) need for the intake of drugs that could affect glucose and lipid metabolism during pregnancy; (4) failure to regularly complete prenatal care or delivery at our hospital, resulting in failure to obtain complete medical records; and (5) gestational age ≥20 weeks at the first antenatal visit. The pregnant women provided written informed consent prior to enrolment. The study protocol was approved by the Ethics Committee of West China Second Hospital of Sichuan University, which follows the principles of the Declaration of Helsinki (2020[073]). This study was registered with the Chinese Clinical Trial Registry (registration number ChiCTR2000041278).

### Research Methods

The HIP screening methods and diagnostic criteria published by the WHO (10) were adopted. Screening was carried out with the 75-g oral glucose tolerance test (OGTT) as follows (11): Prior to the test, a 3-day continuous normal diet was adopted, followed by fasting for ≥ 8 hours before the examination, during which 300 mL of liquid containing 75 g glucose was ingested within 5 minutes. For blood glucose measurement, venous blood was extracted before, and at 1 and 2 hours after sugar intake (counting the time from drinking glucose water). During the examination, the subjects sat quietly and were not permitted to smoke.

GDM was diagnosed if at least one of the following criteria were met (10): (1) FPG ≥5.1 mmol/L but <7.0 mmol/L; (2) 1-hour plasma glucose ≥10.0 mmol/L following a 75-g oral glucose load; and (3) 2-hour plasma glucose ≥8.5 mmol/L but <11.1 mmol/L following a 75-g oral glucose load. DIP was diagnosed if at least one of the following criteria were met: (1) FPG ≥7.0 mmol/L and (2) 2-hour plasma glucose ≥11.1 mmol/L following a 75-g oral glucose load.

**Figure 1:**
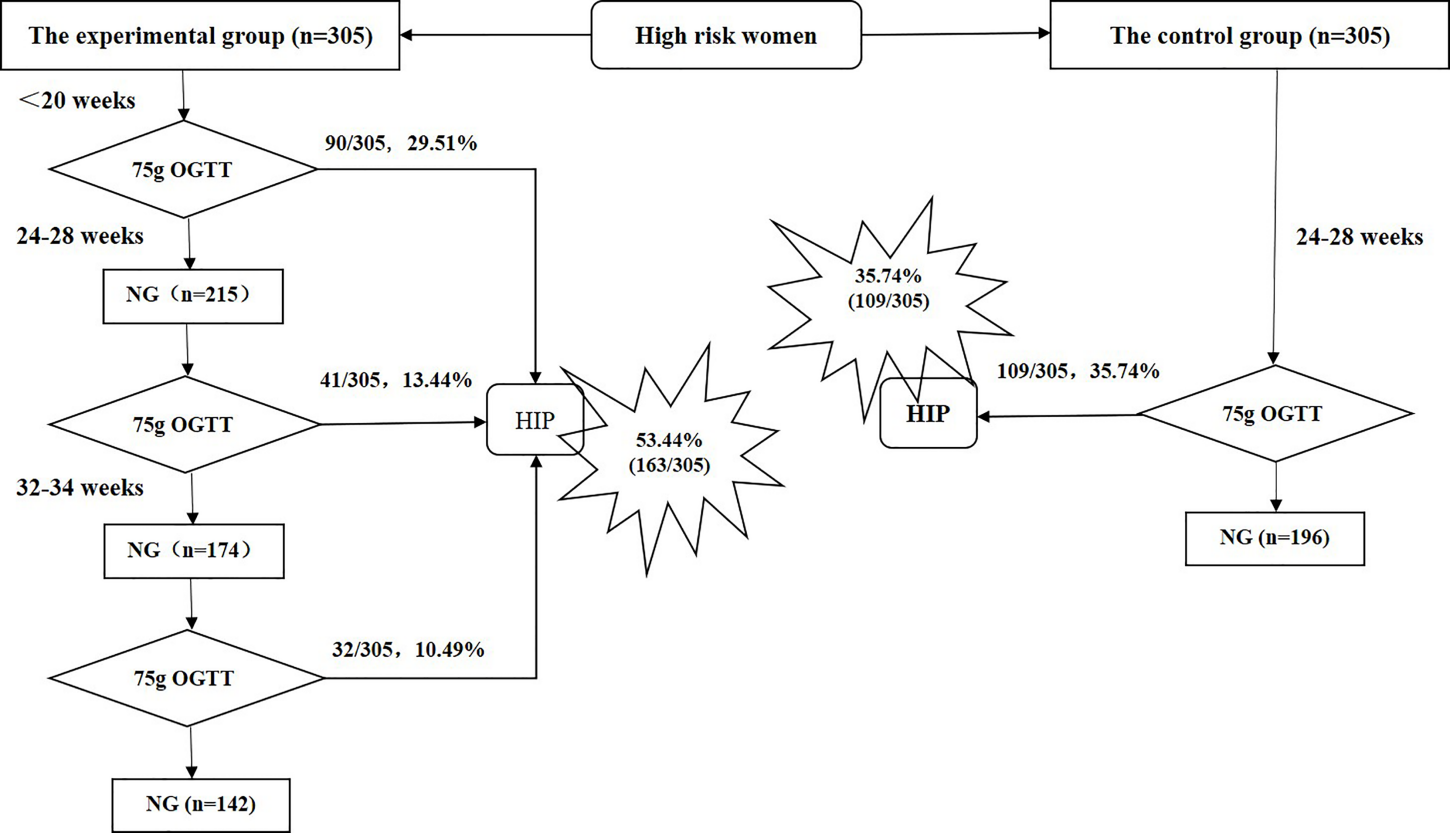
Screening period and hyperglycemia in pregnancy diagnosis in the experimental and control groups. NG, normal glucose; OGTT, oral glucose tolerance test; HIP, hyperglycemia in pregnancy.

Prospective longitudinal sequential studies were only conducted in the experimental group. The pregnant women in the experimental group underwent a 75-g OGTT during their first antenatal visit (early pregnancy, <20 weeks) and in middle (24–28 weeks) and late pregnancy (32–34 weeks); however, those who were diagnosed with HIP did not undergo any subsequent 75-g OGTT. The pregnant women in the control group underwent a 75-g OGTT in middle pregnancy only (24–28 weeks). General information (age, height, pre-pregnancy BMI, gestational age at the first antenatal visit, maternal history, family history, and history of diseases, among others) and perinatal outcome data (gestational age at delivery, birth method, shoulder dystocia, indications for caesarean section, insulin use during pregnancy, pregnancy complications [e.g., hypertensive disorders of pregnancy, premature birth], neonatal weight, Apgar score, incidence of neonatal hypoglycemia, neonatal respiratory distress syndrome, and others) were prospectively collected from the two groups (**Figure 1**).

### Statistical Analysis

Statistical analysis of the data was performed using SPSS 24.0 (IBM Corp., Armonk, NY, USA). Continuous data with a normal distribution and homogeneity of variance were presented as the arithmetic mean ± standard deviation (x ± s). An unpaired *t*-test was used for comparisons between the two groups. Multiple comparisons were conducted using an analysis of variance and were adjusted for *via* the Bonferroni *t*-test. Continuous data without a normal distribution and homogeneity of variance were presented as the median (interquartile range) [M (P25–P75)], and non-parametric tests were used. Categorical variables are presented as counts and percentages, and comparisons between the two groups were performed using the Chi-square test. The Chi-square test for the *R × C* table and the Bonferroni method were employed to adjust for multiple comparisons. Multivariate logistic regression models were used to identify risk factors for diagnosis during different periods. Two-sided p-values <0.05 were considered statistically significant.

## Results

### Comparison of Risk Factors Between the Experimental and Control Groups

Differences in the rates of high-risk factors, such as maternal age ≥35 years, family history of diabetes, pre-pregnancy BMI ≥23 kg/m^2^, PCOS, pre-pregnancy IFG or IGT, previous GDM, and history of macrosomia, were not significant between the experimental and control groups (**Table 1**).

**Table 1:** Comparison of high-risk factors between the experimental and control groups.

High-risk factors	Experimental group (*n*=305)	Control group (*n*=305)	χ^2^	*P*
Maternal age ≥35 years, *n* (%)	156 (51.15)	149 (48.85)	0.321	0.571
Family history of diabetes, *n* (%)	98 (32.13)	92 (30.16)	0.275	0.600
Pre-pregnancy BMI^*^ ≥23 kg/m^2^ *, n* (%)	118 (38.69)	128 (41.97)	0.681	0.409
PCOS^†^, *n* (%)	36 (11.80)	36 (11.80)	0.000	1.000
Pre-pregnancy IFG^‡^ or IGT^§^, *n* (%)	5 (1.64)	1 (0.33)	1.515	0.218
Previous GDM^||^, *n* (%)	40 (13.11)	27 (8.85)	2.834	0.092
History of macrosomia, *n* (%)	19 (6.23)	16 (5.25)	0.273	0.601

^*^BMI, body mass index; ^†^PCOS, polycystic ovary syndrome; ^‡^IFG, impaired fasting glucose; ^§^IGT, impaired glucose tolerance; ^||^GDM, gestational diabetes mellitus.

### HIP Diagnosis in the Experimental and Control Groups

In the experimental group, 163 (53.44%) of 305 women were diagnosed with HIP, among whom 156 (95.71%) had GDM and 7 (4.29%) had DIP. In the control group, 109 (35.74%) of 305 women were diagnosed with HIP, among whom 100 (91.74%) had GDM and 9 (8.26%) had DIP. **Figure 1** shows the numbers of women in the experimental group diagnosed with HIP during early, middle, and late pregnancy. In the experimental group, the overall rate of HIP diagnosis during the early, middle, and late screening periods was significantly higher than that of the control group’s single screening in mid-pregnancy (53.44% vs. 35.74%, *P*<0.001). The rate of HIP diagnosis in the experimental group for the early- and mid-pregnancy screening was higher but not significantly different (42.95% vs. 35.74%, *P*=0.068; **Figure 1**).

A total of 272 women were diagnosed with HIP in the experimental and control groups. Of these, 37 (13.60%) had abnormal FPG levels, 203 (74.63%) had abnormal glucose levels following a 75-g oral glucose load, and 32 (11.76%) had both abnormal FPG levels and abnormal glucose levels following a 75-g oral glucose load. The distributions of the diagnostic basis for HIP during each screening period in the experimental group and during mid-pregnancy screening in the control group were similar to the overall distribution. No significant difference in the distributions of diagnostic basis was observed between the groups (*P*=0.217; **Table 2**).

**Table 2:** Diagnostic basis of HIP diagnosis in different screening periods.

Group	Screening period	A^*^ *n* (%)	B^†^ *n* (%)	A+B*n* (%)	χ^2^	*P*
Experimental group	Early pregnancy (*n*=90)	16 (17.78)	60 (66.67)	14 (15.56)		
	Middle pregnancy (*n*=41)	8 (19.51)	31 (75.61)	2 (4.88)		
	Late pregnancy (*n*=32)	3 (9.38)	25 (78.13)	4 (12.50)		
Control group	Middle pregnancy (*n*=109)	10 (9.17)	87 (79.82)	12 (11.01)		
Total (n=272)		37 (13.60)	203 (74.63)	32 (11.76)	8.168	0.217

^*^Abnormal FPG levels.

^†^Abnormal glucose levels following a 75-g oral glucose load.

### Analysis of Factors Contributing to a Diagnosis of HIP During Early and Late Pregnancy in the Experimental Group

For the experimental group, multivariate logistic regression analysis was used to examine factors influencing the diagnosis of HIP outside the routine screening period (i.e., early and late pregnancy). Data in **Table 3** indicate that previous GDM (odds ratio, OR=9.676, 95% confidence interval [CI]: 4.202–22.279, *P*<0001), pre-pregnancy BMI ≥23 kg/m^2^ (OR=4.273, 95% CI: 2.349–7.773, *P*<0.001), and maternal age ≥35 years (OR=2.377, 95% CI: 1.231–4.590, *P*=0.010) were risk factors for HIP diagnosis in early pregnancy. Previous GDM (OR=8.713, 95% CI: 2.164–35.081, *P*=0.002) was a risk factor for HIP diagnosis in late pregnancy (**Table 4**)Among the 305 subjects in the experimental group, 40 women with previous GDM were identified, among whom 35 (87.50%) were diagnosed with HIP; the diagnosis rates in early, middle, and late pregnancy were 68.57% (24/35), 14.29% (5/35), and 17.14% (6/35), respectively. A total of 118 women had a pre-pregnancy BMI ≥23 kg/m^2^, among whom 64.41% (76/118) were diagnosed with HIP; the diagnosis rates in early, middle, and late pregnancy were 64.47% (49/76), 22.37% (17/76), and 13.16% (10/76), respectively. Among 156 women with maternal age ≥35 years, 53.21% (83/156) were diagnosed with HIP; the diagnosis rates in early, middle, and late pregnancy were 55.42% (46/83), 22.89% (19/83), and 21.69% (18/83), respectively.

**Table 3:** Multivariate logistic regression analysis of factors influencing HIP diagnosis in early pregnancy.

Variables	*B*	Wald χ^2^	*P*	OR	95% CI
Maternal age ≥35 years	0.866	6.654	0.010	2.377	1.231–4.590
Pre-pregnancy BMI ≥23 kg/m^2^	1.452	22.624	<0.001	4.273	2.349–7.773
Family history of diabetes	0.485	2.300	0.129	1.625	0.868–3.042
PCOS^*^	0.640	1.856	0.173	1.897	0.775–4.767
Pre-pregnancy IFG^†^ or IGT^‡^	1.125	1.245	0.264	3.079	0.427–22.193
Previous GDM^§^	2.270	28.448	<0.001	9.676	4.202–22.279
History of macrosomia	-0.119	0.036	0.850	0.888	0.257–3.064

^*^PCOS, polycystic ovary syndrome; ^†^IFG, impaired fasting glucose; ^‡^IGT, impaired glucose tolerance; ^§^GDM, gestational diabetes mellitus.

**Table 4:** Multivariate logistic regression analysis of factors influencing HIP diagnosis in late pregnancy.

Variable	*B*	Wald χ^2^	*P*	OR	95% CI
Maternal age ≥35 years	0.694	1.890	0.169	2.001	0.774–5.380
Pre-pregnancy BMI ≥23 kg/m^2^	0.632	1.567	0.211	1.881	0.700–5.057
Family history of diabetes	0.555	1.235	0.266	1.743	0.654–4.642
PCOS^*^	-0.298	0.112	0.738	0.742	0.129–4.269
Pre-pregnancy IFG^†^ or IGT^‡^	1.359	0.946	0.331	3.892	0.252–60.183
Previous GDM^§^	2.165	9.279	0.002	8.713	2.164–35.081
History of macrosomia	0.582	0.520	0.471	1.790	0.368–8.714

^*^PCOS, polycystic ovary syndrome; ^†^IFG, impaired fasting glucose; ^‡^IGT, impaired glucose tolerance; ^§^GDM, gestational diabetes mellitus.

### Comparison of Perinatal Outcomes

There were no significant differences between the experimental and control groups with respect to perinatal clinical data, including gestational age at delivery, weight gain during pregnancy, neonatal birth weight, or in terms of rates of insulin use, shoulder dystocia, preterm birth, hypertensive disorders of pregnancy, macrosomia, large for gestational age (LGA), low-birth-weight infants, small for gestational age, neonatal respiratory distress syndrome, and neonatal hypoglycemia (**Table 5**).

**Table 5:** Comparison of perinatal outcomes between the experimental and control groups.

Perinatal outcomes	Experimental group (*n*=305)	Control group (*n*=305)	t/χ^2^	*P*
Gestational age at delivery (weeks)	38.77 ± 1.33	38.91 ± 1.30	-1.385	0.167
Weight gain during pregnancy (kg)	11.59 ± 4.45	11.67 ± 4.01	-0.239	0.811
Neonatal birth weight (g)	3284.49 ± 471.01	3281.41 ± 423.77	0.085	0.932
Insulin use, *n* (%)	25 (15.34)	11 (10.09)	0.000	0.994
Shoulder dystocia, *n* (%)	1 (0.33)	0 (0.00)	0.000	1.000
Preterm birth, *n* (%)	21 (6.89)	19 (6.23)	0.107	0.744
Hypertensive disorders of pregnancy, *n* (%)	12 (3.93)	14 (4.59)	0.161	0.689
Polyhydramnios, *n* (%)	5 (1.64)	9 (2.95)	1.170	0.279
Macrosomia, *n* (%)	15 (4.92)	11 (3.61)	0.643	0.423
Large for gestational age, *n* (%)	28 (9.18)	25 (8.20)	0.186	0.666
Low-birth-weight infants, *n* (%)	4 (1.31)	0 (0.00)	2.265	0.132
Small for gestational age, *n* (%)	17 (5.57)	8 (2.62)	3.378	0.660
Neonatal hypoglycemia, *n* (%)	3 (0.98)	3 (0.98)	0.000	1.000
Neonatal respiratory distress syndrome, *n* (%)	2 (0.66)	3 (0.98)	0.000	1.000

The experimental group was thus divided into four subgroups—namely, experimental NBG (*n*=142), experimental early-HIP (*n*=90), experimental middle-HIP (*n*=41), and experimental late-HIP (*n*=32) subgroups. The control group was divided into two subgroups, namely the control NBG (*n*=196) and control middle-HIP (*n*=109) subgroups. The perinatal clinical data of the six subgroups were compared, and the results indicated significant differences in gestational age at delivery (*P*=0.026), weight gain during pregnancy (*P*<0.001), rate of insulin use (*P*=0.031), and incidence of low-birth-weight infants (*P*=0.007) (**Table 6**). We conducted multiple comparisons of these four variables. The gestational age at delivery was significantly earlier in the experimental early-HIP subgroup than in the experimental NBG (38.45 ± 1.61 vs. 38.96 ± 1.30 weeks, *P*=0.04) and control NBG (38.45 ± 1.61 vs. 38.99 ± 1.35 weeks, *P*=0.01) subgroups. Weight gain during pregnancy was significantly lower in the experimental early-HIP subgroup than in the experimental late-HIP (9.65 ± 4.78 vs. 12.09 ± 3.61 kg, *P*=0.003), experimental NBG (9.65 ± 4.78 vs. 13.11 ± 3.87 kg, *P*<0.001), and control NBG (9.65 ± 4.78 vs. 12.52 ± 3.77 kg, *P*<0.001) subgroups. Furthermore, weight gain during pregnancy was significantly lower in the experimental middle-HIP subgroup than in the experimental late-HIP (10.21 ± 4.18 vs. 12.09 ± 3.61 kg, *P*<0.001), experimental NBG (10.21 ± 4.18 vs. 13.11 ± 3.87 kg, *P*=0.048), and control NBG (10.21 ± 4.18 vs. 12.52 ± 3.77 kg, *P*=0.001) subgroups. Weight gain during pregnancy was also significantly lower in the control middle-HIP subgroup than in the experimental late-HIP (10.15 ± 4.01 vs. 12.09 ± 3.61 kg, *P*=0.017), experimental NBG (10.15 ± 4.01 vs. 13.11 ± 3.87 kg, *P*<0.001), and control NBG (10.15 ± 4.01 vs. 12.52 ± 3.77 kg, *P*<0.001) subgroups. However, no significant differences in the rates of insulin use and low-birth-weight infants were noted among these six subgroups after multiple comparisons.

**Table 6:** Comparison of perinatal outcomes among subgroups.

		Gestational age at delivery (week)	Weight gain during pregnancy (kg)	Low-birth-weight infants*n* (%)	Insulin use*n* (%)
	NBG subgroup	38.96 ± 1.30	13.11 ± 3.87	0 (0.00)	-
Experimental group	Early-HIP^*^ subgroup	38.45 ± 1.61	9.65 ± 4.78	3 (2.11)	19 (13.38)
	Middle-HIP^*^ subgroup	38.71 ± 1.03	10.21 ± 4.18	0 (0.00)	5 (3.52)
	Late-HIP^*^ subgroup	38.84 ± 0.64	12.09 ± 3.61	1 (0.70)	1 (0.70)
Control group	NBG subgroup	38.99 ± 1.35	12.52 ± 3.77	0 (0.00)	-
	Middle-HIP^*^ subgroup	38.77 ± 1.20	10.15 ± 4.01	0 (0.00)	11 (7.75)
t/χ^2^		2.568	14.241	10.441	8.686
*P*		0.026	<0.001	0.007	0.031

^*^HIP, hyperglycemia in pregnancy.

## Discussion

### Diagnosis of HIP in High-Risk Pregnant Women

Few studies have conducted sequential screening in early, middle, and late pregnancy for women with high-risk factors for HIP. In our experimental group, the HIP diagnosis rate was approximately 54% (163/305); among these patients, almost 30% were diagnosed early at the first antenatal visit, and a further 10% were diagnosed at the conventional screening point in mid-pregnancy. More importantly, the screening strategy identified an additional 10% of HIP cases in late pregnancy. The percentages of pregnant women diagnosed with HIP were 55.21%, 25.15%, and 19.63% in the three screening periods, respectively, confirming the value of additional screening in early and late pregnancy. The study similar to ours was a multi-country, multicenter study from Europe that included only pregnant women with pre-pregnancy obesity and analyzed HIP diagnosis at <20, 24–28, and 35–37 weeks. In that study, the overall incidence was approximately 39% (395/1023), whereas the incidence at <20, 24–28, and 35–37 gestational weeks was 23.66% (242/1023), 9.19% (94/1023), and 5.77% (59/1023), respectively. Among 395 subjects diagnosed with HIP, the rates of diagnosis were 61.27%, 23.80%, and 14.94% in early, middle, and late pregnancy, respectively (5). The screening method employed and distribution of overall diagnoses in that study were similar to those in ours, although it only involved one risk factor and had partial loss to follow-up in middle and late pregnancy.

Most related studies have focused on comparing early to routine screening. A meta-analysis (12) revealed that 15–70% of high-risk pregnant women were diagnosed with GDM during early screening. Bianchi et al. (13) recruited 290 pregnant women with prior GDM, pre-pregnancy BMI ≥30 kg/m^2^, or FPG levels of 5.55–6.94 mmol/L at the first prenatal visit; half had undergone sequential screening in early and middle pregnancy (experimental group), whereas the other half had been conventionally screened in middle pregnancy (control group). They reported similar overall HIP diagnosis rates between the experimental and control groups, but in the experimental group, 42.7% of high-risk women had been diagnosed in early pregnancy, and 31.3% of women with normal 75-g OGTT results in early pregnancy had been diagnosed in their second trimester. Thus, the overall diagnosis rate of sequential screening in early and middle pregnancy was consistent with that of routine screening in middle pregnancy; nevertheless, more than half of high-risk pregnant women had hyperglycaemia prior to middle pregnancy and had been diagnosed in early pregnancy, which is similar to our results. Bozkurt et al. (14) investigated the pathophysiological characteristics of pregnant women with GDM and reported that pregnant women with GDM diagnosed in early pregnancy had lower insulin sensitivity and abnormal cell function than those with GDM diagnosed in middle pregnancy or those with NBG levels. Immanuel J et al. (15), investigated the physiological composition of GDM in early gestation, they found that insulin resistance contributed relatively more than reduced insulin secretion to the development of early GDM in this mainly obese population and was associated with greater risk of adverse perinatal outcomes. Based on these studies, we speculate that women could benefit from screening for insulin resistance in early pregnancy, but there were no measures of insulin resistance and secretion in our study, which needs to be improved in subsequent studies.

Few studies have employed repeat OGTT in late pregnancy. A Dutch study (16) reported that 23.5% of pregnant women were diagnosed with GDM based on a second OGTT in late pregnancy. In our study, 174 pregnant women in the experimental group who had normal OGTT results in the first and middle periods underwent a repeat OGTT in late pregnancy, and 32 (18.39%) of the 174 pregnant women received a supplementary diagnosis, with the diagnosis rates being similar in middle and late pregnancy (13.44% vs 10.49%). However, considering the few reported findings and weak evidence of late-pregnancy screening, HIP diagnosis in late-pregnancy still requires further research.

### High-Risk Factors and Screening Period

Domestic and international guidelines include numerous risk factors for HIP, such as specific race (e.g., Asian), advanced age, being overweight or obese before pregnancy, family history of diabetes, and previous GDM. Nevertheless, numerous studies have shown that these risk factors are not identical (17, 18). When early OGTT in high-risk pregnant women yields normal results, OGTT in subsequent middle pregnancy is required and may be repeated in the third trimester in some women. Considering the inconvenience and discomfort of OGTT, it is of great importance to analyze the correlation between risk factors and the diagnosis period to further narrow the target population and achieve “precise” screening. The European multicenter study (19) mentioned above also analyzed this issue and found clinical characteristics independently associated with GDM/overt diabetes differed by OGTT time point. The identified risk factors can help define the target population for future intervention trials.

A study (6) reported that previous GDM (OR=22.3, 95% CI: 12.2–40.7) and class-III obesity (OR=12.7, 95% CI: 10.3–15.6) were most strongly associated with early diagnosis. Other influencing factors included class-II obesity (OR=6.85, 95% CI: 5.64–8.33), class-I obesity (OR=3.3, 95% CI: 2.77–3.93), overweight (OR=1.35, 95% CI: 1.14–1.61), maternal age ≥35 years (OR=1.24, 95% CI: 1.06–1.46), and diabetes in a first-degree relative (OR=1.50, 95% CI: 1.31–40.7). Another European study (20) showed that GDM in early pregnancy was associated with previous GDM (OR=2.74, 95% CI: 1.66–4.50), history of delivering a large infant (OR=1.97, 95% CI: 1.03–3.98), and a higher pre-pregnancy BMI (OR=1.05, 95% CI: 1.00–1.10). Our results indicated that previous GDM, pre-pregnancy BMI ≥23 kg/m^2^, and maternal age ≥35 years were risk factors for HIP diagnosis during the first prenatal visit and that previous GDM was also a risk factor for HIP diagnosis in late pregnancy.

Several studies have shown the role of previous GDM in early HIP diagnosis. The recurrence of hyperglycaemia in subsequent pregnancies indicates the recurrence of GDM may even be due to abnormal glucose metabolism before subsequent pregnancies. In a meta-analysis (21) that included 18 studies enrolling 19,053 women, the overall recurrence rate was 48% for GDM, and the subgroup analysis indicated higher recurrence rates of 59% and 54% for Hispanic and Asian women, respectively. Here, the overall recurrence rate for GDM in 67 pregnant women with previous GDM was 74.63% (50/67) and was as high as 87.5% (35/40) in the experimental group. Furthermore, 24 (68.57%), 5 (14.28%), and 6 (17.14%) of 35 pregnant women with recurrent GDM in the experimental group were diagnosed in early, middle, and late pregnancy, respectively. This not only shows that sequential screening programs in early, middle, and late pregnancy may screen more women with recurrent GDM, but also indicates that more than two-thirds of women with recurrent GDM may be diagnosed early in the first prenatal test, and that nearly one-fifth of women may receive supplementary diagnosis in the third trimester. Additionally, this study found that previous GDM was a risk factor for HIP diagnosis in late pregnancy. Despite few relevant studies, the correlation between previous GDM and HIP diagnosis in late pregnancy (OR=2.6, 95% CI: 1.0–6.3) has been reported (16).

Different populations have different associations with BMI, body fat percentage, and body fat distribution. According to the WHO and evidence from studies conducted in several Asian countries, 23 kg/m^2^ can be used as the cut-off point for overweight in the Asian population (22). The guidelines for GDM by the American College of Obstetricians and Gynecologists (23) regard overweight or obesity as a necessary condition, fully emphasizing the importance of BMI. These guidelines also note that Asian women with a BMI ≥23 kg/m^2^ are considered overweight. Therefore, pre-pregnancy BMI ≥23 kg/m^2^ was included as a high-risk factor in our study. Overweight or obesity is a common risk factor associated with early diagnosis (6, 20). In our study, HIP was diagnosed in 64.41% (76/118) of high-risk pregnant women with a pre-pregnancy BMI ≥23 kg/m^2^ in the experimental group, and most were diagnosed at the first antenatal visit (64.47%, 49/76). Harreiter J et al. (20) reported that early GDM was significantly more common with higher pre-pregnancy BMI, the sum of skinfolds in early pregnancy, prior history of GDM, and previous macrosomia, and in a subanalysis of nulliparous women, multivariate logistic regression found that higher prepregnancy BMI was the only risk factor for an early GDM diagnosis. Early screening of overweight and obese pregnant women in early pregnancy may attract the attention of both doctors and patients, and weight and blood glucose management may be carried out in the early period.

We identified that maternal age ≥35 years was a risk factor for HIP diagnosis at the first antenatal visit, which has also been previously reported. Here, more than half of high-risk pregnant women aged ≥35 years were diagnosed with HIP, and 55% of them were diagnosed at the first antenatal visit. In the context of comprehensive implementation of the two-child policy in China, the number of older pregnant women is increasing, and we should, therefore, pay attention to early HIP screening in this group.

### Selection of Screening Methods for High-Risk Pregnant Women

The global guidelines recommend HIP screening for pregnant women with risk factors at the first prenatal visit or in early pregnancy; however, recommendations for screening methods vary. The Society of Obstetricians and Gynecologists of Canada (24) consider that both a 50-g glucose challenge test and 75-g OGTT may be used for early pregnancy screening. The Canadian Diabetes Association (25) indicates that non-pregnancy screening tests (FPG or HbA1c) or 75-g OGTT may be used in early pregnancy. However, the National Institute for Health and Care Excellence (26) clearly suggests that 75-g OGTT should be used instead of FPG, random blood glucose, HbA1c, glucose challenge test, or urine sugar to test for HIP in women with risk factors. According to the Queensland guidelines (27), FPG may only reflect part of glucose metabolism, whereas HbA1c is only suitable for diagnostic testing in early pregnancy. Nonetheless, the principal value of HbA1c lies in the identification of women who likely have pre-existing glucose abnormalities rather than milder degrees of hyperglycaemia and known hemoglobinopathies; moreover, the effects on HbA1c results should be considered. Guidelines offer little advice on selecting appropriate screening methods for late pregnancy. Our results showed that less than one-fifth of pregnant women were diagnosed with HIP by a simple abnormality in FPG levels at different stages of pregnancy, and most of them presented with abnormal blood glucose levels following a 75-g oral glucose load. Therefore, irrespective of whether the OGTT is conducted during the routine screening period or in early or late pregnancy, it is a reasonable and necessary choice because it may detect any degree of hyperglycaemia and reflects both fasting and post-load blood glucose levels. However, our result may not apply to all areas, because in one European research (20), fasting glucose alone identified 78.5% of women in early pregnancy, and 21.5% were diagnosed with elevated 1-h and/or 2-h glucose levels.

### Influence of the Screening Strategy on Perinatal Outcomes

HIP is closely related to long- and short-term adverse maternal and infant outcomes (28), and effective management is essential for reducing adverse consequences. We look forward to early pregnancy screening to ensure earlier treatment as well as additional screening to avoid misdiagnosis to reduce adverse outcomes due to non-intervention. However, studies have drawn mixed conclusions concerning the relationship between these screening strategies and perinatal outcomes. A study (29) confirmed that early screening improved the primary composite outcome (emergency caesarean section, neonatal hypoglycemia, and macrosomia; OR=0.62, 95% CI: 0.43–0.91). Nevertheless, several studies have not arrived at that conclusion. Hosseini et al. (30) compared pregnant women with abnormal blood glucose levels to those with NBG levels during pregnancy. They reported that diagnosis in early pregnancy was associated with an increased risk of macrosomia, LGA, caesarean section, 1-minute Apgar score <7 among neonates, neonatal respiratory distress syndrome, and admission to the neonatal intensive care unit, whereas diagnosis in middle pregnancy was only associated with an increased risk of macrosomia, LGA, and caesarean section. Similarly, Harper et al. (31) conducted a trial involving 962 obese women and concluded that early screening did not reduce the incidence of the primary composite outcome (macrosomia, shoulder dystocia, neonatal hypoglycemia, neonatal hyperbilirubinemia). This may be related to the fact that diagnosis in early pregnancy denotes the more serious pathological status of these pregnant women (13). Our results indicated no significant difference in the incidence of adverse outcomes between the experimental and control groups. However, in a paired comparison of subgroups, we observed that the weight gain during pregnancy was lower in the experimental early-HIP, usual-middle-HIP, and control NBG subgroups than in the experimental late-HIP, experimental NBG, and control NBG subgroups, suggesting that early diagnosis aids in capturing the attention of both doctors and pregnant women, thus facilitating pregnancy weight management.

In summary, the impact of early screening on perinatal outcomes cannot be easily concluded, and further research is required. Furthermore, no study has reported the influence of early diagnosis on long-term maternal and pediatric outcomes; hence, this field also needs to be explored. Few studies investigating late pregnancy screening have been conducted. A study (16) reported that the rates of macrosomia (16.3% vs. 35.3%, *P*=0.011) and LGA (18.6% vs. 39.7%, *P*=0.018) were lower in individuals with abnormal blood glucose levels than in those with NBG levels in late pregnancy. We did not show that supplementary diagnosis in late pregnancy improved the short-term perinatal outcomes.

This is the first study on a sequential screening strategy in early, middle, and late pregnancy for women with HIP risk, which identified the role of sequential screening strategy in increasing HIP diagnosis rates in high-risk pregnant women and identified high-risk pregnant women who needed additional screening and tried to explore the impact of the new screening strategy on perinatal outcomes. Our study makes up for the gap in screening HIP in pregnant women at high risk and provides a reference value for obstetricians in clinical work. But our study is limited because we only explored the effects on maternal and infant perinatal outcomes. As we know, the effects of the HIP do not end with the end of pregnancy. Considering the influence of HIP on the mother and her offspring, further research is warranted to explore the long-term outcomes of high-risk pregnant women and their newborns, which is what our team is currently working on.

In conclusion, we showed that sequential screening in early, middle, and late pregnancy significantly increased the HIP diagnosis rate in high-risk pregnant women. The earlier the diagnosis, the more likely both doctors and patients would focus on weight gain control during pregnancy. Previous GDM, pre-pregnancy BMI ≥23 kg/m^2^, and maternal age ≥35 years were risk factors for HIP diagnosis at the first antenatal visit, whereas previous GDM was a risk factor for HIP diagnosis in late pregnancy. Less than one-fifth of pregnant women were diagnosed with HIP by simple abnormality in FPG levels at different stages of pregnancy, and most presented with abnormal blood glucose levels following a 75-g oral glucose load. Therefore, 75-g OGTT is a reasonable and necessary choice for pregnancy screening in high-risk pregnant women. We did not observe beneficial effects of sequential screening in early, middle, and late pregnancy on perinatal outcomes other than weight control during pregnancy. Nonetheless, considering all treatment-related costs, it may represent a preventive investment against type 2 DM, cardiovascular disorders, and kidney diseases for the mother and against type 2 DM and metabolic syndrome for her offspring. Consequently, we recommend sequential screening in early and middle pregnancy for high-risk pregnant women with maternal age ≥35 years or pre-pregnancy BMI ≥23 kg/m^2^ and in early, middle, and late pregnancy for high-risk pregnant women with previous GDM.

## Data Availability Statement

The original contributions presented in the study are included in the article/**Supplementary Material**. Further inquiries can be directed to the corresponding author.

## Ethics Statement

The studies involving human participants were reviewed and approved by West China Second University Hospital, Sichuan University. The patients/participants provided their written informed consent to participate in this study.

## Author Contributions

LZ is the guarantor of this work and, as such, had full access to all the data in the study and takes responsibility for the integrity of the data and the accuracy of the data analysis. YX drafted the manuscript and collected and analyzed the data. LZ conceived and designed the study, helped draft and critically revise the manuscript, and coordinated the data collection. QW contributed to the data collection and analysis and helped draft the manuscript. M-FD, Y-MW, and NH contributed to the data collection and analysis. All authors read and approved the final manuscript.

## Funding

This research was supported by the Sichuan Health and Welfare Commission of Popularization Project (grant number 18PJ063), the Science and Technology Foundation of Sichuan Province (grant number 2019YFS0410) and the Clinical Research Foundation of West China Second Hospital of Sichuan University.

## Conflict of Interest

The authors declare that the research was conducted in the absence of any commercial or financial relationships that could be construed as a potential conflict of interest.

## Publisher’s Note

All claims expressed in this article are solely those of the authors and do not necessarily represent those of their affiliated organizations, or those of the publisher, the editors and the reviewers. Any product that may be evaluated in this article, or claim that may be made by its manufacturer, is not guaranteed or endorsed by the publisher.

## Abbreviations

DIP, diabetes in pregnancy; FPG, fasting plasma glucose; GDM, gestational diabetes mellitus; HAPO, Hyperglycemia and Adverse Pregnancy Outcome; HIP, hyperglycaemia in pregnancy; IADPSG, International Association of Diabetes and Pregnancy Study Groups; IGT, impaired glucose tolerance; LGA, large for gestational age; NBG, normal blood glucose; PCOS, polycystic ovary syndrome.
