# Supplementary material for: Sequential Screening Strategy in Early, Middle, and Late Pregnancy in Women at High Risk of Hyperglycemia
Source: Front Endocrinol (Lausanne). 2022 Jun 6;13:829388. doi: 10.3389/fendo.2022.829388 (PMC9207315; doi:10.3389/fendo.2022.829388)
Supplement: Supplementary file 1 [file DataSheet_1.docx]

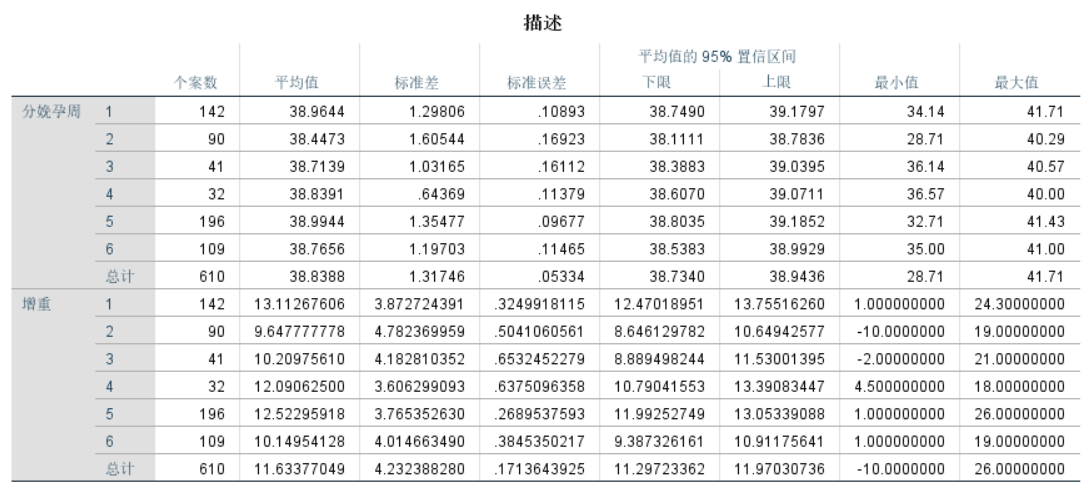


Mean

Weight gain during pregnancy

1-experimental NBG; 2- experimental early-HIP subgroup; 3- experimental middle-HIP subgroup; 4- experimental late-HIP;

5- control NBG; 6- control middle-HIP subgroup

The gestational age at delivery

The gestational age at delivery


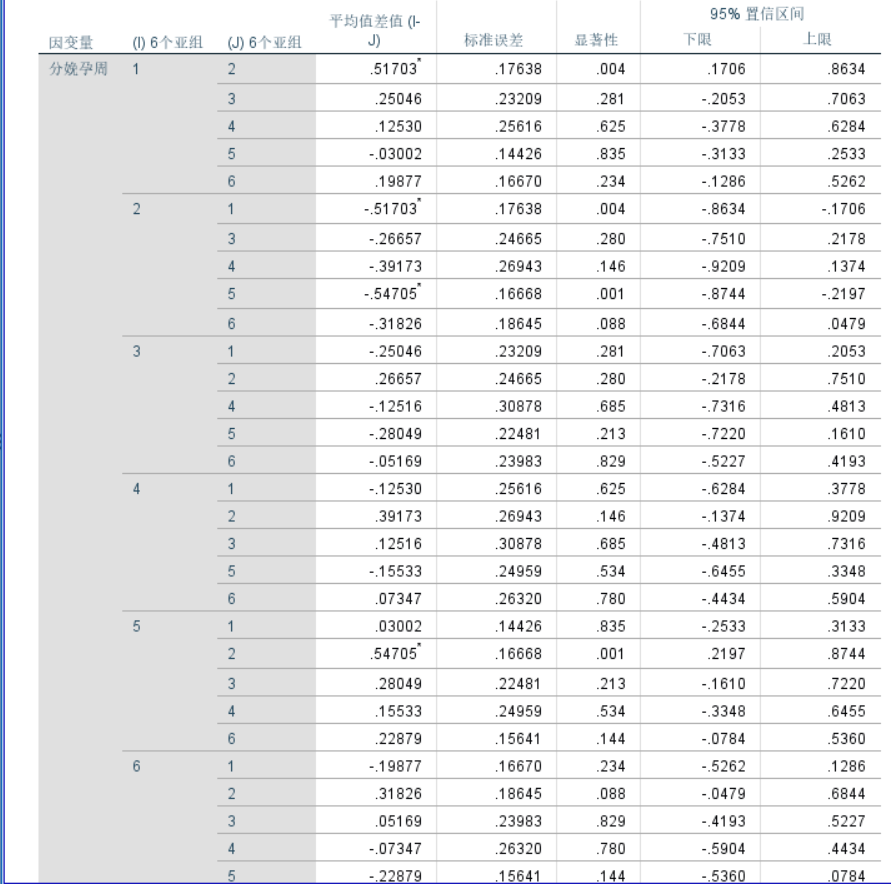


1-experimental NBG; 2- experimental early-HIP subgroup; 3- experimental middle-HIP subgroup; 4- experimental late-HIP;

5- control NBG; 6- control middle-HIP subgroup


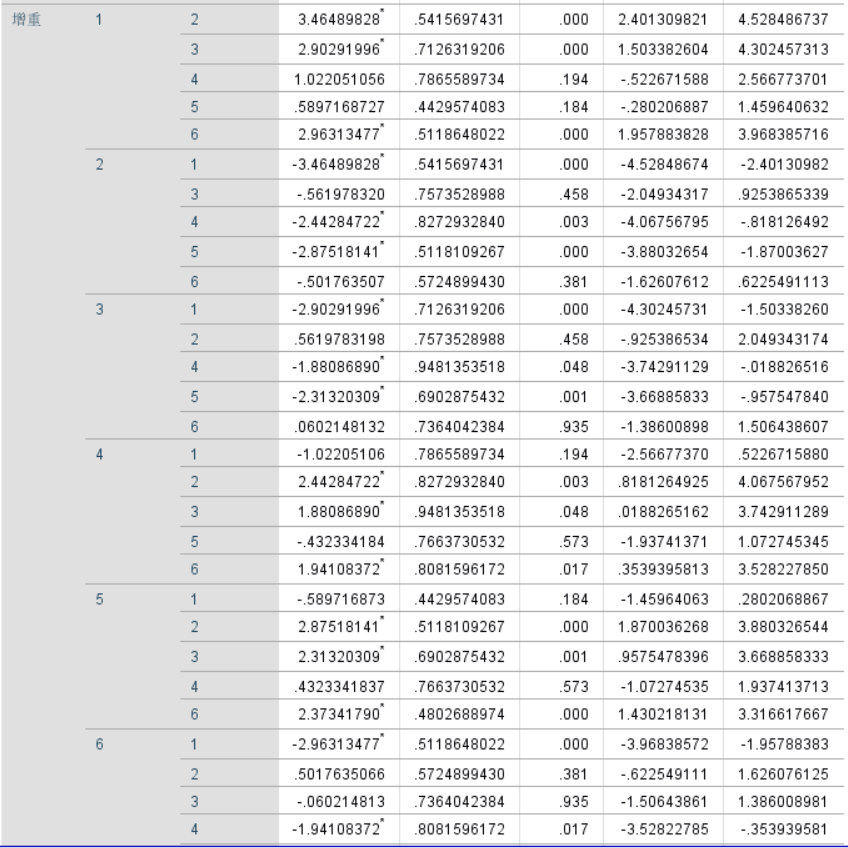


Weight gain during pregnancy

1-experimental NBG; 2- experimental early-HIP subgroup; 3- experimental middle-HIP subgroup; 4- experimental late-HIP;

5- control NBG; 6- control middle-HIP subgroup
